# Oxidative profiling of the failing right heart in rats with pulmonary hypertension

**DOI:** 10.1371/journal.pone.0176887

**Published:** 2017-05-04

**Authors:** Xinhong Wang, Nataliia V. Shults, Yuichiro J. Suzuki

**Affiliations:** 1 Department of Pharmacology and Physiology, Georgetown University Medical Center, Washington, DC, United States of America; 2 Department of Physiology and Pathophysiology, School of Basic Medical Sciences, Fudan University, Shanghai, China; Augusta University, UNITED STATES

## Abstract

Right heart failure is the major cause of death among patients with pulmonary arterial hypertension (PAH). Understanding the biology of the right ventricle (RV) should help developing new therapeutic strategies. Rats subjected to the injection of Sugen5416 (an inhibitors of vascular endothelial growth factor receptor) plus the ovalbumin immunization had increased pulmonary arterial pressure and severe vascular remodeling. RVs of these rats were hypertrophied and had severe cardiac fibrosis. No apoptosis was, however, detected. Metabolomics analysis revealed that oxidized glutathione, xanthine and uric acid had increased in PAH RVs, suggesting the production of reactive oxygen species by xanthine oxidase. PAH RVs were also found to have a 30-fold lower level of α-tocopherol nicotinate, consistent with oxidative stress decreasing antioxidants and also demonstrating for the first time that the nicotinate ester of vitamin E is endogenously expressed. Oxidative/nitrosative protein modifications including *S*-glutathionylation, *S*-nitrosylation and nitrotyrosine formation, but not protein carbonylation, were found to be increased in RVs of rats with PAH. Mass spectrometry identified that *S*-nitrosylated proteins include heat shock protein 90 and sarcoplasmic reticulum Ca^2+^-ATPase. These results demonstrate that RV failure is associated with the promotion of specific oxidative and nitrosative stress.

## Introduction

Pulmonary arterial hypertension (PAH) affects both men and women of any age, including children. PAH is characterized by increased blood pressure in the pulmonary circulation with abnormal pathologies in the pulmonary vessels. Despite the availability of approved drugs for treatment, PAH remains a fatal disease [1–4]. Increased pulmonary artery (PA) resistance results in right-sided heart failure and death [5–8]. The median reported survival for PAH patients is 2.8 years from the time of diagnosis (3-yr survival of 48%) if left untreated [3,9]. Even with currently available therapies, prognosis remains poor, with 3-yr survival of 58–75% [10–12]. Thus, new and improved therapeutic strategies are needed to satisfactorily treat PAH patients.

Right heart failure is the major cause of death among patients with PAH [13]. The mortality of PAH patients who are admitted to intensive care unit with RV failure is 41% [14]. However, the pathophysiology of the right heart has not been well defined [15]. The mechanism of right heart failure in response to pulmonary hypertension and that of left heart failure in response to systemic hypertension appear to be quite different. In the left ventricle (LV), concentric hypertrophy transitions to dilation with eccentric cardiomyocyte hypertrophy and thinning of the ventricular wall. In contrast, right heat failure in *cor pulmonale* exhibits a concentrically hypertrophied right ventricle (RV) [16,17]. Further, the LV and RV originate from different progenitor cells [18,19]. Thus, understanding how the RV is altered in response to pulmonary hypertension should help developing right heart-specific therapeutic strategies to better manage PAH patients [20].

While it remains yet undetermined whether or not the information obtained in animal models translates to human pathophysiology, RVs of various pulmonary hypertension models including chronic hypoxia, monocrotaline, and Sugen5416/hypoxia have been studied in order to provide a foundation for understanding the RV biology. It is, however, unclear whether these animals ultimately die of RV failure. Recently, Voelkel and co-workers developed an experimental model of PAH in rats through the injection of Sugen5416 (a small molecule inhibitor of VEGF receptor/tyrosine kinase) and ovalbumin (OVA) immunization [21]. This model exhibits the development of severe angio-obliterative PAH with 20% of the animals dying due to RV failure within 8 weeks [21,22]. Thus, in addition to studying the role of inflammation in the pathogenesis of PAH, the OVA/Sugen5416 model may present a suitable system to study the pathology of RV failure. The present study characterized, in detail, the lungs and RVs of rats subjected to OVA and Sugen5416.

## Materials and methods

### Animal treatment

**Fig 1:**
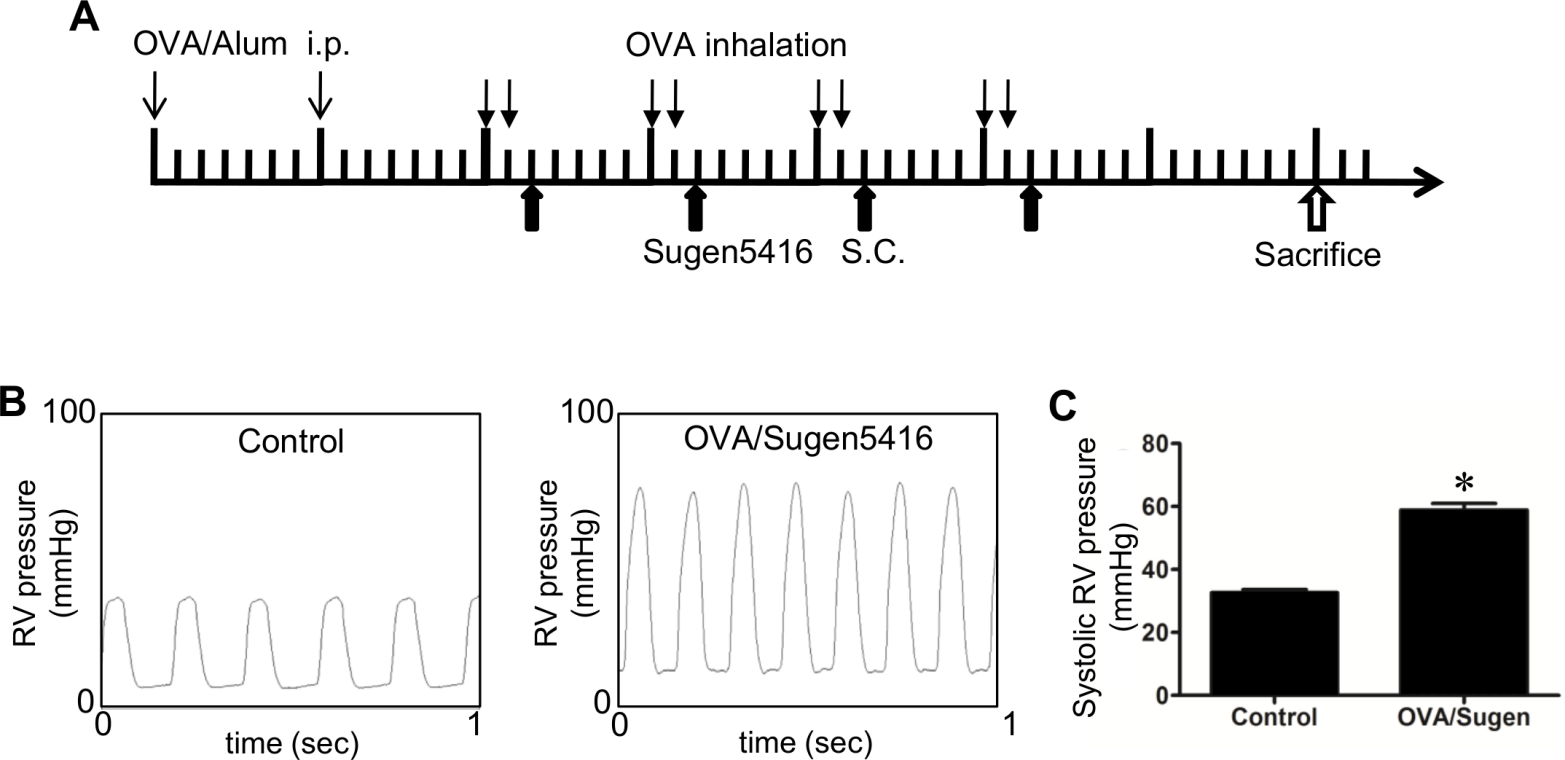
Hemodynamics characteristics of the OVA/Sugen5416 model of PAH. (A) Schematics of the rat model of PAH induced by OVA and Sugen5416. Rats were injected intraperitoneally on Days 1 and 8 with 1 mg of OVA with Imject Alum Adjuvant (OVA/Alum) for sensitization. On Day 15, rats were subjected to 30 min of 1% OVA inhalation with a nebulizer, which continued twice a week for four weeks. Sugen5416 (20 mg/kg body weight) was injected subcutaneously (S.C.) once a week on the day after OVA inhalation. Hemodynamic measurements were performed, rats were sacrificed, and lung and heart tissues were obtained on Day 50. (B) Representative traces of hemodynamic measurements taken from the RV using a Millar catheter. (C) The bar graph represents means ± SEM of systolic RV pressure. * denotes a value that is significantly different from the control value at *P* < 0.05 (N = 6).

PAH was induced by administering OVA and Sugen5416 to male Sprague-Dawley rats (Charles River Laboratories, Wilmington, MA, USA) using a protocol previously described^21^ (Fig 1A). At the end of the experiments, rats were anesthetized by the intraperitoneal injection of urethane (1.6 g/kg body weight). They were then intubated and mechanically ventilated using a volume-controlled Inspira Advanced Safety Ventilator (Harvard Apparatus, Holliston, MA, USA). Rats were kept on a heat pad to maintain temperature at 37°C using a TR-200 Temperature Controller connected to a rectal probe (Fine Scientific Tools, North Vancouver, Canada). After thoracotomy, a Millar catheter (1.4 F) was inserted into the RV, and RV pressure was recorded using PowerLab with Chart 5 software (AD Instruments, Colorado Springs, CO, USA) [23].

The Georgetown University Animal Care and Use Committee approved all animal experiments, and the investigation conformed to the National Institutes of Health Guide for the Care and Use of Laboratory Animals. A total of 24 rats were used in this study.

### Histological measurements

Tissues were immersed in buffered 10% formalin at room temperature and embedded in paraffin. Paraffin-embedded tissues were cut and mounted on glass slides. Tissue sections were subjected to hematoxylin and eosin (H&E) stain, Verhoeff-van Gieson stain, Masson’s trichrome stain, and immunohistochemistry with the α-smooth muscle actin antibody (Abcam, Cambridge, MA, USA).

### Western blot analysis

Tissues were minced and homogenized, and protein gel electrophoresis samples were prepared [23]. For Western blotting, equal protein amounts (as determined by the Bradford method, Bio-Rad Laboratories, Hercules, CA) of either homogenate or immunoprecipitated samples were electrophoresed through an SDS polyacrylamide gel and then electroblotted onto a nitrocellulose membrane (Bio-Rad). The membrane was blocked and incubated with antibodies against alpha-1 type I collagen, glutathione, nitrotyrosine, heat shock protein 90 (HSP90), cardiac sarco/endoplasmic reticulum Ca^2+^-ATPase (SERCA2) (Santa Cruz Biotechnology, Dallas, TX, USA), *S*-nitrosocysteine, malondialdehyde (MDA), 4-hydroxynonenal (4-HNE) (Alpha Diagnostics Intl. Inc., San Antonio, TX, USA), and protein carbonyls (by using OxyBlot; EMD Millipore, Billerica, MA, USA). Levels of proteins were detected using horseradish peroxidase-linked secondary antibodies (Bio-Rad) and an Enhanced Chemiluminescence System (GE Healthcare Bio-Sciences, Pittsburgh, PA, USA). SulfoBiotics Protein *S*-Nitrosylation Monitoring Kit (Dojindo Molecular Technologies, Rockville, MD, USA) was used to determine the number of *S*-nitrosylated cysteine residues.

### Metabolomics

RV tissues were homogenized in 50% methanol containing internal standards and then centrifuged at 13,000 rpm for 10 min. An equal volume of chilled acetonitrile was added to each sample tube, vortexed and incubated overnight at -20°C. Tubes were centrifuged at 13,000 rpm for 10 min at room temperature; and the supernatant was transferred to fresh tubes and dried under vacuum. The dried metabolite mixture was resuspended in 100 uL of 50% methanol for mass spectrometry analysis.

Samples were injected onto a reverse-phase column using an Acquity ultra-performance liquid chromatography (UPLC) system (Waters Corporation, USA). Mass spectrometry was performed using a Quadrupole-time-of-flight mass spectrometer operating in either negative or positive electrospray ionization mode with a capillary voltage of 3.2 kV and a sampling cone voltage of 35 V. Data were acquired in centroid mode with a mass range from 50 to 850 m/z for TOF-MS scanning. Duplicates (technical replicates) of each sample were tested in positive and negative ionization modes to determine the chromatographic reproducibility.

### Mass spectrometry for protein identification

Protein bands were excised from the Coomassie Blue-stained gels. Mass spectra were recorded using a matrix-assisted laser desorption/ionization–time-of-flight, time-of-flight (MALDI-TOF-TOF) spectrometer (4800 Proteomics Analyzer, Framingham, MA, USA). Peptide masses were compared with the theoretical masses derived from the sequences contained in SWISS-PROT/NCBI databases using MASCOT [24].

### Statistical analysis

Means and standard errors were calculated. Comparisons between the two groups were analyzed using a two-tailed Student’s *t* test. *P* < 0.05 was considered significant.

## Results

### OVA/Sugen5416 promotes pulmonary hypertension and pulmonary vascular remodeling

Rats were treated with the OVA immunization plus the Sugen5416 injection, as described in Fig 1A. Seven weeks after the first injection of OVA, hemodynamic measurements were performed in anaesthetized, open-chested rats by inserting a Millar catheter into the RV apex. Fig 1B shows representative traces of the hemodynamic measurements from control rats and from rats treated with OVA/Sugen5416. This treatment produced pulmonary hypertension, with systolic RV pressure elevated to ~60 mmHg (Fig 1C). Neither heart rate nor body weight was significantly altered by the Sugen5416 plus OVA treatment.

**Fig 2:**
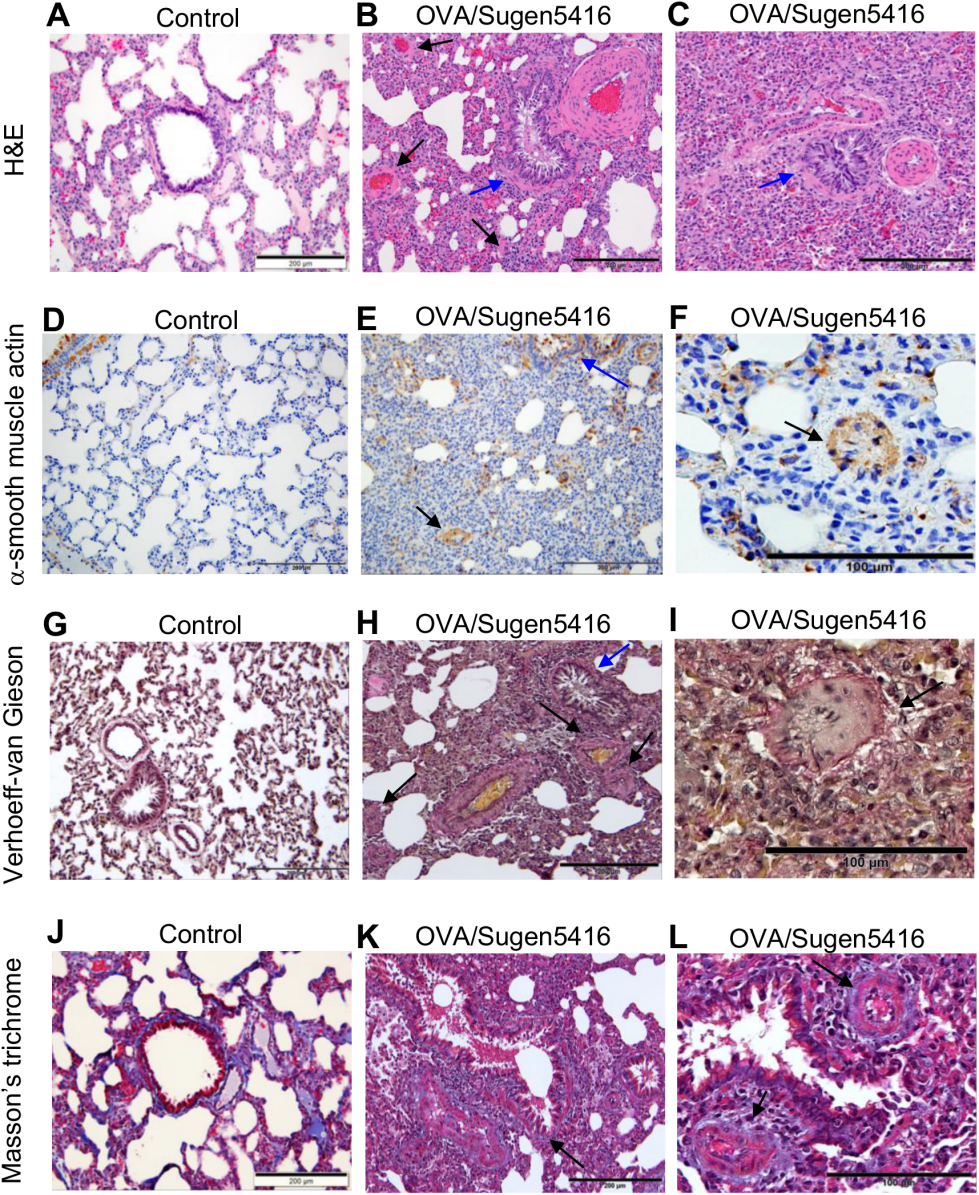
Histological characterizations of the lungs of rats treated with OVA/Sugen5416. Rats were subjected to treatment with OVA and Sugen5416 to promote PAH. Paraffin-embedded lung sections were subjected to (A—C) H&E stain, (D—F) immunohistochemistry with the α-smooth muscle actin antibody, (G–I) Verhoeff-van Gieson stain and (J—L) Masson’s trichrome stain.

Histological evaluations of H&E-stained lung sections revealed that, compared to control rats (Fig 2A), rats subjected to OVA/Sugen5416 exhibited severe pulmonary vascular remodeling (Fig 2B & 2C). Pulmonary vessel wall thickened; the vascular lumen was nearly or completely occluded; and perivascular inflammatory infiltrates occurred (arrows in Fig 2B). The lungs of OVA/Sugen5416-treated rats also showed atelectasis and the emphysematous expansion of alveoli, with the alveolar septa being thickened with inflammatory infiltrates (Fig 2B & 2C). Treatment with OVA also seems to have caused airway remodeling, since bronchial walls are thickened, with observable swelling. The bronchiolar epithelium is hypertrophied with the hyperplasia of secretory cells (blue arrows in Fig 2B & 2C). Bronchial spasms are apparent and the bronchial lumen is either dramatically reduced as a result of mucus secretion or completely occluded (Fig 2C).

Immunohistochemistry using the antibody against α-smooth muscle actin (Fig 2D–2F) demonstrated increased medial thickness of the PA (black arrows in Fig 2E & 2F) as well as hypertrophy of the muscular layer of the bronchial wall (blue arrow in Fig 2E).

Verhoeff-Van Gieson stain shows that, compared to normal lungs (Fig 2G), lungs from rats treated with OVA/Sugen5416 have concentric, intimal and medial thickening of the PA, with increased levels of collagen and elastic fibers occluding the lumen and fibrous tissues surrounding the vessels (black arrows in Fig 2H & 2I). The bronchial epithelium exhibits mucous hyperplasia and hyper-secretion with significant thickening of the basement membrane and the bronchiolar wall as a result of increased collagen fibers and peribronchial fibrosis (blue arrow in Fig 2H). Masson’s trichrome stain of the lungs from OVA/Sugen5416-treated rats also shows collagen fibers occurring in the pulmonary interstitium; a homogenous thickening of the epithelial reticular basement membrane of the airway wall; and peribrochiolar fibrosis (arrow in Fig 2K). Fig 2L also shows concentric, laminar intimal fibrosis (arrows); concentric narrowing of the pulmonary arterial lumen as a result of collagen increased in the medial layer; and complete luminal occlusion of the small PA. Fibrous tissues surrounding both PAs and bronchioles are also observed.

### RV hypertrophy and fibrosis in rats with PAH

**Fig 3:**
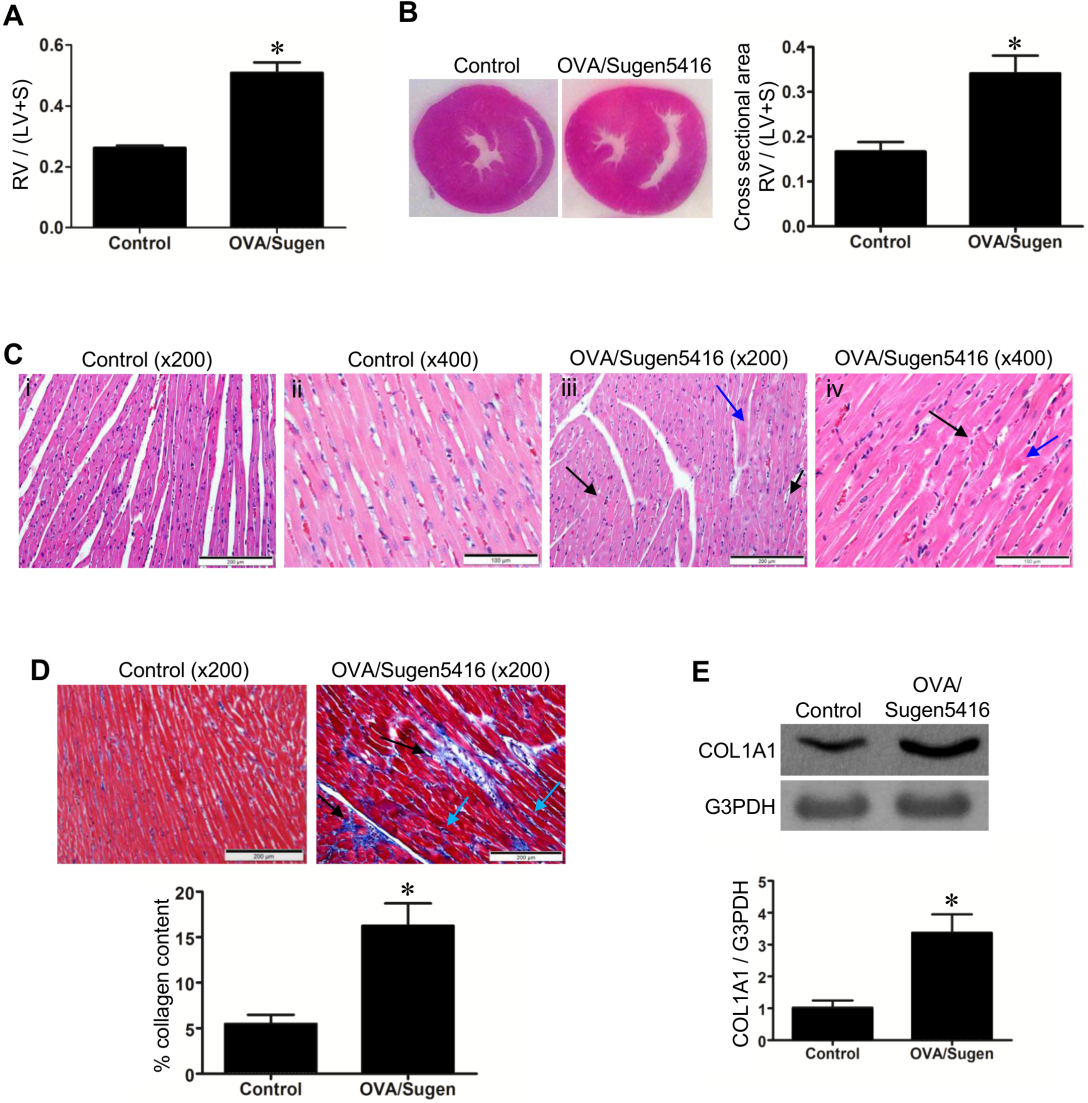
RV changes in rats with PAH. Rats were subjected to treatment with OVA and Sugen5416 to promote PAH. (A) Fulton ratio, the ratio of RV mass / (LV mass + septum mass). (B) H&E stain of the heart sections. The bar graph represents the ratio of the cross-sectional areas of RV / (LV + septum). (C) Microscopic observations of the H&E-stained RV. (i & ii) The longitudinal section of a representative control RV under 200x and 400x magnifications. (iii & iv) The longitudinal section of a representative RV from PAH rats under 200x and 400x magnifications. (D) Masson’s trichrome stain was performed on RV sections. Muscle fibers stain red. Collagen stains blue. (E) RV homogenates were subjected to western blotting to detect alpha-1 type I collagen (COL1A1) as an indicator of fibrosis. Bar graphs represent means ± SEM. * denote values significantly different from controls at *P* < 0.05 (N = 6).

RVs of rats with PAH, due to OVA/Sugen5416, exhibit cardiac hypertrophy with the mass increasing twofold as indicated by Fulton index measurements (Fig 3A) and visualized in H&E staining of the heart (Fig 3B). Microscopic observations of H&E-stained heart sections identified the syncytium of myocardial fibers with central nuclei in the RVs of control rats (Fig 3C i & ii). In OVA/Sugen5416-treated rats with PAH, by contrast, myocyte degeneration; polymorphic cardiomyocytes; hypertrophy of cardiomyocytes; myofiber disarray; hypereosinophilia and hyperbasophilia of cardiomyocytes (black arrows in Fig 3C iii); and focal lesions of cardiomyocytes (blue arrow in Fig 3C iii) were observed. Further, in RVs of PAH animals, contractures of cardiomyocytes (black arrow); myocytolysis (blue arrow); loss of myofiber striations; wavy arrangement of myofiber; edema of cytoplasm; and indistinct nuclear borders of cardiomyocytes were observed (Fig 3C iv).

Treated RVs also suffered from cardiac fibrosis, as visualized by Masson’s trichrome-stained heart sections (Fig 3D) and assessed by the expression of alpha-1 type I collagen by Western blotting of RV homogenates (Fig 3E). Black arrows in Fig 3D shows the PAH RV exhibits replacement fibrosis in the form of microfocal areas replacing altered cardiomyocytes. Perivascular fibrosis and diffused interstitial fibrosis (blue arrows in Fig 3D) are also present in the RV of OVA/Sugen5416-treated rats.

### Metabolomics studies of the RV

**Fig 4:**
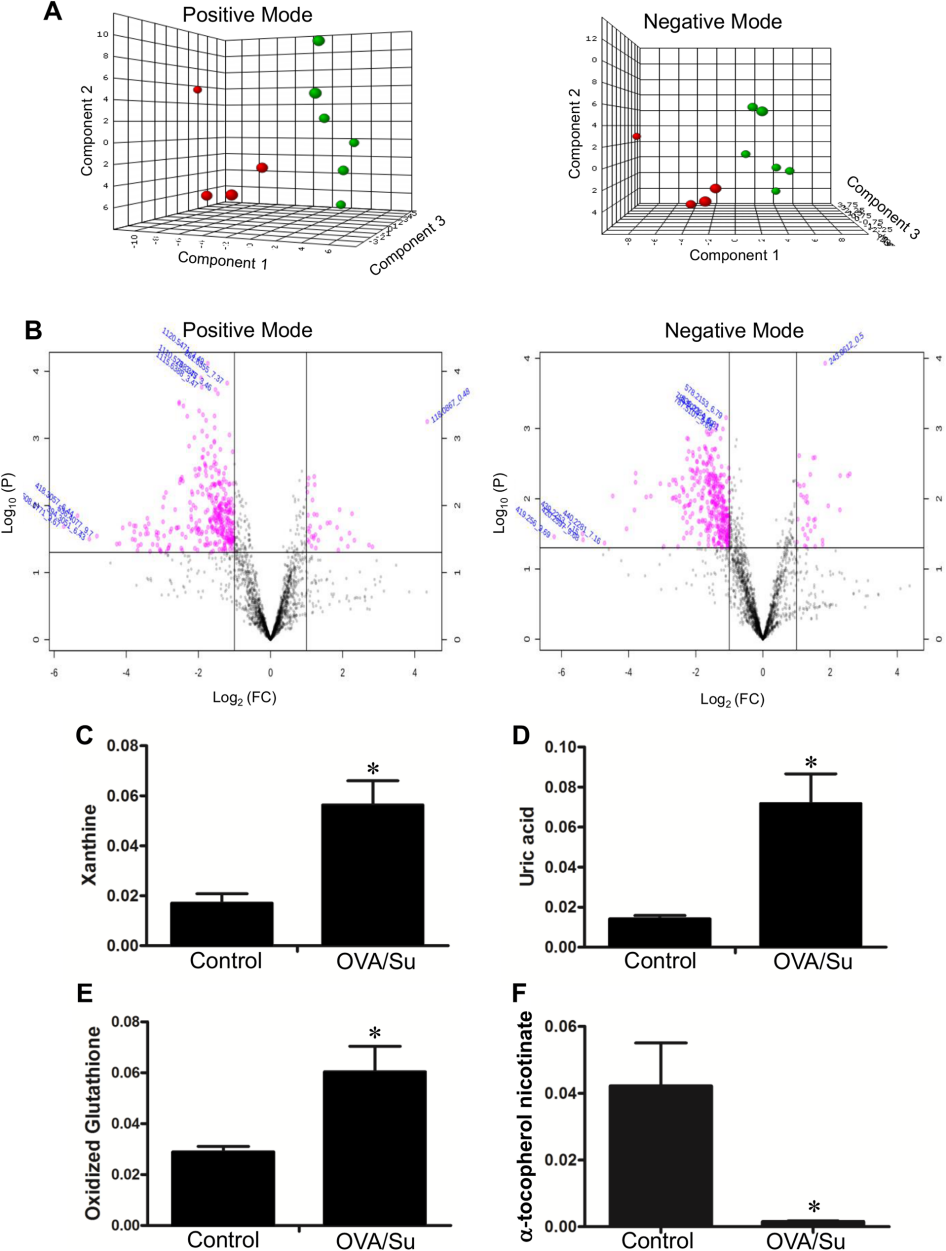
Metabolomics studies of the RV. Metabolomics analysis were performed in RV tissues from control rats and rats subjected to OVA/Sugen5416 to promote PAH. (A) PLS-DA plots for positive and negative modes of ionization with a 95% confidence region for OVA/Sugen5416 (green circle; N = 6) vs. control (red circle; N = 4). (B) Volcano plot in positive and negative ionization modes for OVA/Sugen5416 vs. control. Dot plots represent fold changes (FC) on the x-axis and the statistical significance in *P* value (P) on the y-axis. Pink dots represent values with *P* ≤ 0.05 and a multiplicative change of greater than twofold. (C) Xanthine, (D) uric acid, (E) oxidized glutathione and (F) α-tocopherol nicotinate levels in control rat RVs and RVs of rats treated with OVA/Sugen5416 (OVA/Su). Bar graphs represent means ± SEM. * denote values that are significantly different from control at *P* < 0.05.

To obtain further understanding of the RVs of rats with PAH via the administration of OVA and Sugen5416, metabolomics analysis was performed using UPLC and mass spectrometry to determine metabolite profiles. The partial least squares—discriminatory analysis (PLS-DA) plots suggest that PAH RVs and control RVs have distinct metabolomics profiles in positive and negative ionization modes (Fig 4A). Pink dots shown in the Volcano plots represent metabolites whose levels were found to be significantly different at *P* < 0.05 with ≥ 2-fold changes (Fig 4B). Notably, RVs of rats treated with OVA/Sugen5416 had a 3.8-fold increase in xanthine (m/z 153.04; *P* = 0.012; Fig 4C) and 4.9-fold increase in uric acid (m/z 167.02; *P* = 0.014; Fig 4D). Since both of these molecules are enzymatic products of xanthine oxidase, the results suggest that this enzyme is activated in RVs of PAH rats. In the two sequential xanthine oxidase reactions which use hypoxanthine and xanthine as substrates, molecular oxygen is reduced to a reactive oxygen species (ROS), the superoxide anion radical. Metabolomics results also identified that PAH caused a 2.1-fold increase in oxidized glutathione (m/z 613.16; *P* = 0.035; Fig 4E) and a 28.2-fold decrease in α-tocopherol nicotinate (m/z 536.41; *P* = 0.029; Fig 4F) in the RV, consistent with the production of ROS.

### Oxidative and nitrosative protein modifications in the RVs of rats with PAH

**Fig 5:**
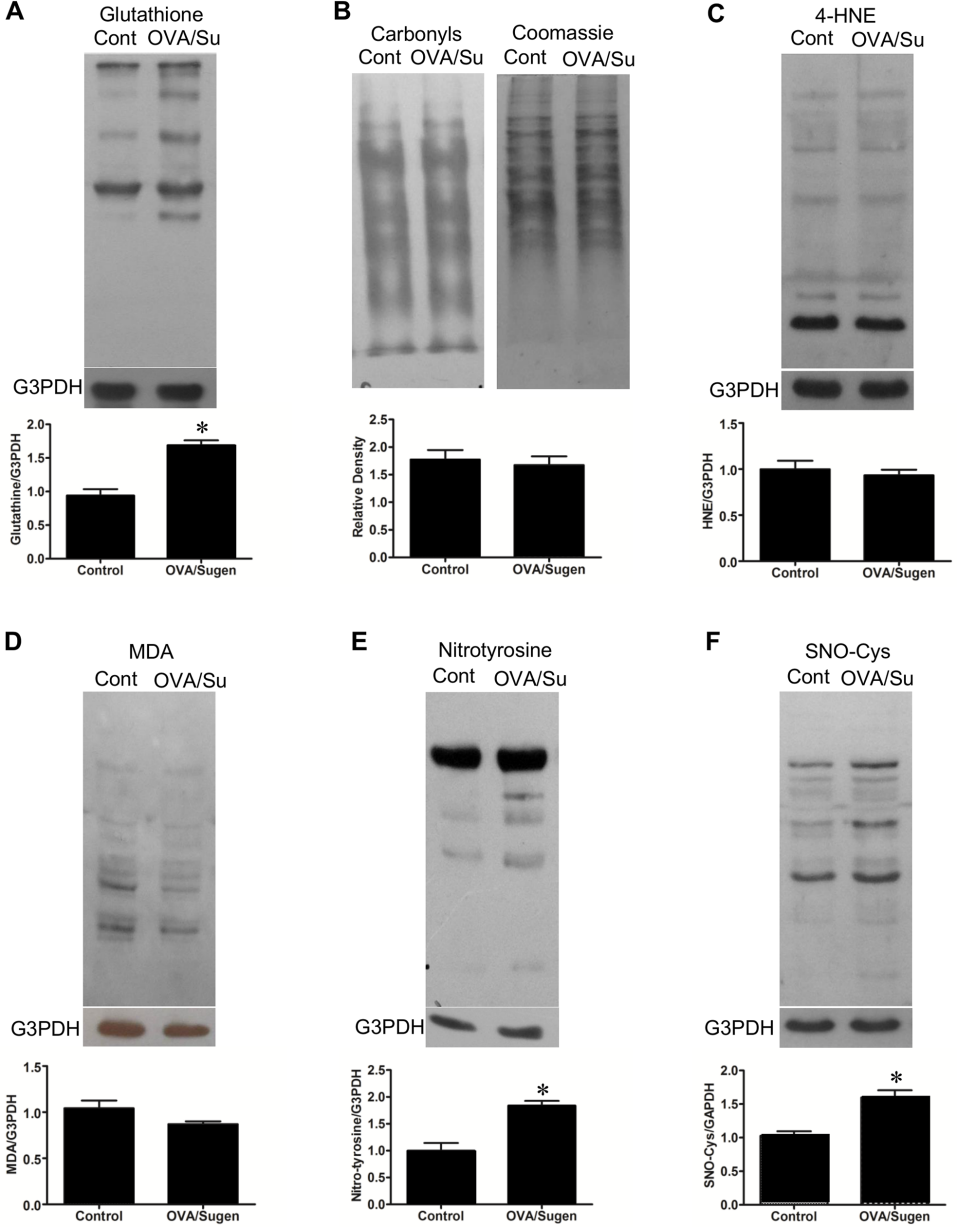
Oxidative/nitrosative protein modification in the RVs of rats with PAH. RV homogenates prepared from control rats (Cont) and rats treated with OVA and Sugen5416 (OVA/Su) to promote PAH were subjected to Western blotting with (A) the glutathione antibody to detect *S*-glutathionylation, (B) the antibody against 2,4-dinitrophenylhydrazine to detect protein carbonyls, (C) 4-hydroxylnonenal (4-HNE) antibody, (D) malondialdehyde (MDA) antibody, (E) nitrotyrosine antibody and (F) nitrosocysteine antibody. Bar graphs represent means ± SEM. * denote values significantly different from controls at *P* < 0.05 (N = 6).

Since metabolomics analysis indicated the production of ROS, oxidative modifications of proteins were monitored by Western blotting. Consistent with the production of superoxide radicals that are converted to hydrogen peroxide as well as the increased oxidized glutathione level as revealed by metabolomics studies described above, RVs of OVA/Sugen5416-treated rats had increased *S*-glutathionylated proteins (Fig 5A) that are produced by hydrogen peroxide. Hydrogen peroxide also gets reduced to the hydroxyl radical via metal-catalyzed oxidation, however, hydroxyl radical-dependent protein oxidation products, protein carbonyls as monitored using 2,4-dinitrophenylhydrazine, were not increased in RVs of PAH animals (Fig 5B). Similarly, protein adducts with lipid peroxidation products, 4-hydroxynonenal (Fig 5C) or malondialdehyde (Fig 5D) that are often formed via hydroxyl radicals were not increased. Another important oxidative protein modification is the nitrotyrosine formation that is formed by interactions of protein tyrosine residues with peroxynitrite that is produced by the reaction between superoxide and nitric oxide. As shown in Fig 5E, the total nitrotyrosine formation of various proteins was higher in the RVs of PAH animals compared to controls. These results indicate that the nitric oxide production is promoted in RVs of OVA/Sugen5416-treated rats. Consistently, protein *S*-nitrosylation that is formed by the reaction of protein cysteine residues with nitric oxide are also higher in PAH RVs (Fig 5F).

**Fig 6:**
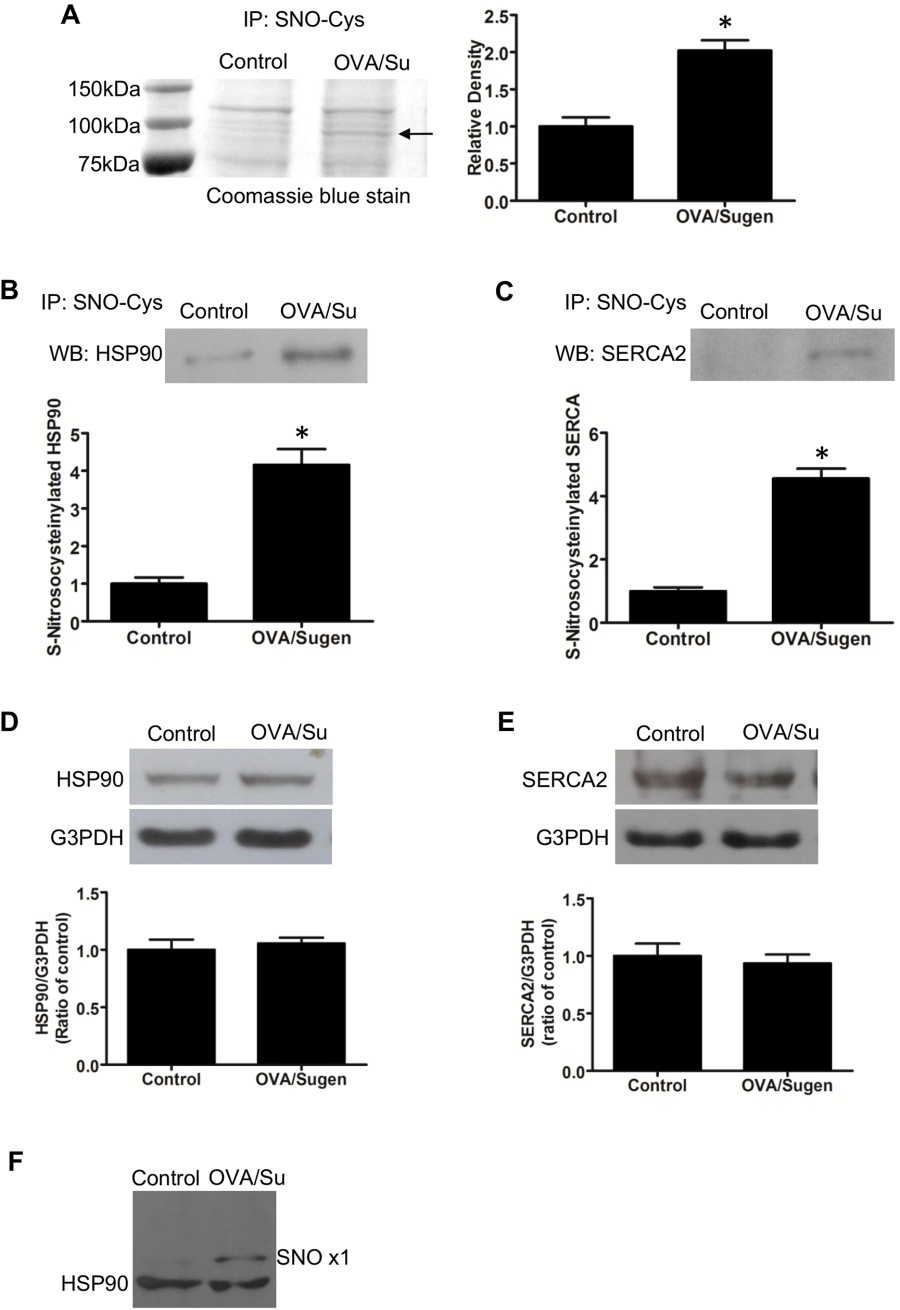
Protein *S*-nitrosylation in the RVs of rats with PAH. (A) RV homogenates prepared from control rats (Cont) and rats treated with OVA and Sugen5416 (OVA/Su) to promote PAH were immunoprecipitated with the antibody against *S*-nitrosocysteine (SNO-Cys). Immunoprecipitated samples were subjected to SDS-PAGE and stained with Coomassie Blue. The band indicated by an arrow, which was consistently higher in OVA/Sugen5416-treated (OVA/Su) animals, was analyzed by mass spectrometry. (B & C) Mass spectrometry identifications of HSP90 and SERCA2 were confirmed by subjecting immunoprecipitated samples to Western blotting. (D & E) Western blotting showing that protein expression levels of HSP90 and SERCA2 are not different between RVs of control rats and those of rats with PAH. Bar graphs represent means ± SEM. * denote values significantly different from controls at *P* < 0.05 (N = 6). (F) RV homogenates prepared from control rats and rats treated with OVA/Sugen5416 were subjected to Dojindo SulfoBiotics Protein *S*-Nitrosylation Monitoring assay and subsequently to Western blotting with the HSP90 antibody. SNO x1 depicts HSP90 with one cysteine residue nitrosylated.

We attempted to identify proteins that are modified in RVs of PAH animals. We found a ~100 kDa band that contains *S*-nitrosylated proteins in PAH RVs (Fig 6A). Mass spectrometry identified heat shock protein (HSP90) and cardiac sarco/endoplasmic reticulum Ca^2+^-ATPase (SERCA2) are in this band. Immunoprecipitation with the nitrosocysteine antibody followed by Western blotting with the HSP90 antibody (Fig 6B) or the SERCA2 antibody (Fig 6C) also indicated that these proteins are *S*-nitrosylated in response to Ova/Sugen5416-dependent PAH in the RV. Total protein expression levels of neither HSP90 (Fig 6D) nor SERCA2 (Fig 6E) were altered in RVs in response to OVA/Sugen5416 treatment.

*S*-nitrosylation of HSP90 in RVs of OVA/Sugen5416-treated rats was confirmed by using the SulfoBiotics Protein *S*-Nitrosylation Monitoring System that was developed by Dojindo Molecular Technologies. In this system, free sulfhydryl groups of cysteine residues are blocked, *S*-nitrosylated thiols are reduced, and the resultant free thiols are labeled with Protein-Shifter Plus which adds ~15 kDa to proteins per *S*-nitrosylated cysteine residue. Samples were then subjected to Western blotting with the HSP90 antibody. As shown in Fig 6F, control RV homogenates exhibit the 90kDa HSP90 band in the first lane. The second lane with OVA/Su5416 RV homogenates has, in addition to the band of HSP90 that is not *S*-nitrosylated, a ~105kDa band that depicts HSP90 with one cysteine residue being *S*-nitrosylated.

## Discussion

We studied the lungs and RVs of rats subjected to OVA immunization and Sugen5416 injection. Mizuno et al. [21] previously reported that this model exhibits increased PA pressure, PA remodeling and RV hypertrophy. Our histological examination further revealed that this PAH model is associated with airway remodeling and atelectasis. In the RV, various features of cardiac pathology including fibrosis were observed.

Oxidative stress has been implicated in various pathophysiological conditions [25]. Similarly, RVs of some animal models of PAH have been shown to be accompanied by increased oxidative stress. In RVs, the production of ROS was observed in monocrotaline-injected rats [26], by PA constriction in mice [27,28] and in rats treated with Sugen5416/hypoxia [29,30]. Our study showed that oxidized glutathione is increased in the RVs of the OVA/Sugen5416 model. Whether the antioxidant treatment attenuates the development of RV failure by scavenging RV ROS is not yet known, because the only study that used a reliable antioxidant molecule, EUK-134 (a SOD and catalase mimetic) cannot distinguish whether this antioxidant affected the ROS that were involved in the development of pulmonary hypertension or RV failure [31].

Mechanistic insights on the role of ROS in RV failure were obtained in the present study, which showed that RVs of rats treated with OVA/Sugen5416 exhibited oxidative and nitrosative protein modifications through *S*-glutathionylation, nitrotyrosine formation and *S*-nitrosylation without the apparent promotion of protein carbonylation. These differential oxidative protein modifications can be explained by considering the role of iron since the formation of both primary and secondary protein carbonylation is Fenton reaction-dependent [25,32]. To our knowledge, this is the first demonstration of the occurrence of the cysteine modifications including *S*-glutathionylation and *S*-nitrosylation in RV failure.

Our mass spectrometry and immunological studies revealed that HSP90 and SERCA2 are the targets of *S*-nitrosylation. HSP90 maintains proteostasis. SERCA2 regulates Ca^2+^ homeostasis. Both of these proteins have been implicated in heart failure [33,34]. Thus, inhibition of these proteins via cysteine modifications is expected to play important roles in PAH-induced RV alterations and possibly right heart failure.

ROS appear to be produced in the RV in response to pulmonary hypertension by multiple sources. NADPH oxidase has been suggested as a mechanism of the increased ROS production in the RV of the monocrotaline model [26]. In our study, metabolomics analysis identified increased levels of xanthine and uric acid, indicating that RVs of PAH animals have increased xanthine oxidase activity.

A large decrease in α-tocopherol nicotinate (a vitamin E ester of niacin) was also revealed by the metabolomics analysis. A decrease in this antioxidant is consistent with the excessive production of ROS. Further, we demonstrate, for the first time, that this esterified form of vitamin E is endogenous expressed and gets altered in disease states. Further examination of α-tocopherol nicotinate, which has not been studied, may provide important information on mechanisms of diseases, including that of right heart failure in response to PAH.

## Conclusions

The present study investigated the properties of RVs in rats with PAH induced by the OVA immunization and Sugen5416 injection. The lungs of these animals have both pulmonary vascular remodeling and airway remodeling that may occur when asthma and pulmonary hypertension coexist in patients. This rat model appears to exhibit RV failure that is similar to human right heart failure. Using this model, the present study advanced the understanding of oxidative and nitrosative stress in PAH-induced RV alterations, opening up future investigations for assessing redox biology of RV failure.

## Abbreviations

4-HNE: 4-hydroxynonenal
H&E: hematoxylin and eosin
HSP90: heat shock protein 90
LV: left ventricle
MDA: malondialdehyde
OVA: ovalbumin
PA: pulmonary artery
PAH: pulmonary arterial hypertension
ROS: reactive oxygen species
RV: right ventricle
SERCA: sarco/endoplasmic reticulum Ca^2+^-ATPase
